# Low-energy shock waves evoke intracellular Ca^2+^ increases independently of sonoporation

**DOI:** 10.1038/s41598-019-39806-x

**Published:** 2019-03-01

**Authors:** Toru Takahashi, Keiichi Nakagawa, Shigeru Tada, Akira Tsukamoto

**Affiliations:** 1 Department of Applied Physics, Graduate School of Science and Engineering, National Defense Academy, Hashirimizu 1-10-20, Yokosuka, Kanagawa 239-8686 Japan; 2 Department of Precise Engineering, Graduate School of Engineering, The University of Tokyo, Hongo 7-3-1, Bunkyo-ku, Tokyo 113-8656 Japan

## Abstract

Low-energy shock waves (LESWs) accelerate the healing of a broad range of tissue injuries, including angiogenesis and bone fractures. In cells, LESW irradiations enhance gene expression and protein synthesis. One probable mechanism underlying the enhancements is mechanosensing. Shock waves also can induce sonoporation. Thus, sonoporation is another probable mechanism underlying the enhancements. It remains elusive whether LESWs require sonoporation to evoke cellular responses. An intracellular Ca^2+^ increase was evoked with LESW irradiations in endothelial cells. The minimum acoustic energy required for sufficient evocation was 1.7 μJ/mm^2^. With the same acoustic energy, sonoporation, by which calcein and propidium iodide would become permeated, was not observed. It was found that intracellular Ca^2+^ increases evoked by LESW irradiations do not require sonoporation. In the intracellular Ca^2+^ increase, actin cytoskeletons and stretch-activated Ca^2+^ channels were involved; however, microtubules were not. In addition, with Ca^2+^ influx through the Ca^2+^ channels, the Ca^2+^ release through the PLC-IP_3_-IP_3_R cascade contributed to the intracellular Ca^2+^ increase. These results demonstrate that LESW irradiations can evoke cellular responses independently of sonoporation. Rather, LESW irradiations evoke cellular responses through mechanosensing.

## Introduction

Shock waves were first applied in medicine as extracorporeal shock wave therapies (ESWT) in the early 1980 s. A decade later, shock waves were found to affect treatments for musculoskeletal disorders^1,2^. Recently, shock waves have been further applied to heal wounds, burns, and ischemia^3–8^. The acoustic energies of shock waves that are irradiated for tissue healing are limited to a tenth of the energies of shock waves used for ESWT^7^. Therefore, shock waves for tissue healing are termed low-energy shock waves (LESWs). LESW irradiations enhance gene expression and protein synthesis in cells^9–12^. Mechanosensors such as VE-cadherin, caveolin, and integrin are involved in the enhancements. Thus, mechanosensing is supposed to be involved in cellular signals evoked by LESW irradiations.

Sonoporation is a formation of transient pores on plasma membrane caused by the application of acoustic energies on cells^13^. Foreign genes and drugs are transmitted into cytosol through transient pores on plasma membrane. Irradiating shock waves cause sonoporation in cells^14,15^. Through the transient pores formed by shock waves, intracellular biomolecules could be released into extracellular space. Those released biomolecules can evoke cellular signals in pore-formed cells themselves or in neighboring cells. From this point of view, sonoporation is expected to be another possible mechanism underlying cellular signals induced by LESW irradiations^9,10^. However, whether sonoporation is involved in evoking cellular signals remains elusive.

Intracellular Ca^2+^ increase is a second messenger that regulates broad cellular fates^16,17^. This second messenger is often evoked by various mechanical stimulations including mechanical stretch and shear stress^18–22^. Fluorescence imaging reveals the intracellular Ca^2+^ increase in single cells. This single-cell detection enables the detection of subtle cellular responses in a limited number of cells. The mechanical stimulation required to evoke such a subtle cellular response should be weaker than that required to evoke a large number of cells. In previous studies, cellular responses those were evoked by LESW irradiations were detected as a result of enhancements of gene expression and protein syntheses. Those detections require large numbers of cells. By detecting an intracellular Ca^2+^ increase, it is expected that the acoustic energy of shock waves required to detect a cellular response could be suppressed. When acoustic energy is suppressed, sonoporation is also suppressed^15^. With suppressed acoustic energy, an intracellular Ca^2+^ increase is expected to be evoked under conditions in which sonoporation is suppressed or excluded.

In this study, intracellular Ca^2+^ increases were observed in endothelial cells on which LESWs were irradiated with spatially uniform acoustic energies. The acoustic energy necessary to evoke intracellular Ca^2+^ increases was quantified. Sonoporation was detected in cells on which LESWs were irradiated with the minimum acoustic energy in order to understand whether the minimum acoustic energy is also sufficient for sonoporation. In cells in which an intracellular Ca^2+^ increase was evoked with the minimum acoustic energy, cascades underlying the evocation were further investigated.

## Results

### LESWs evoke intracellular Ca^2+^ increase

**Figure 1:**
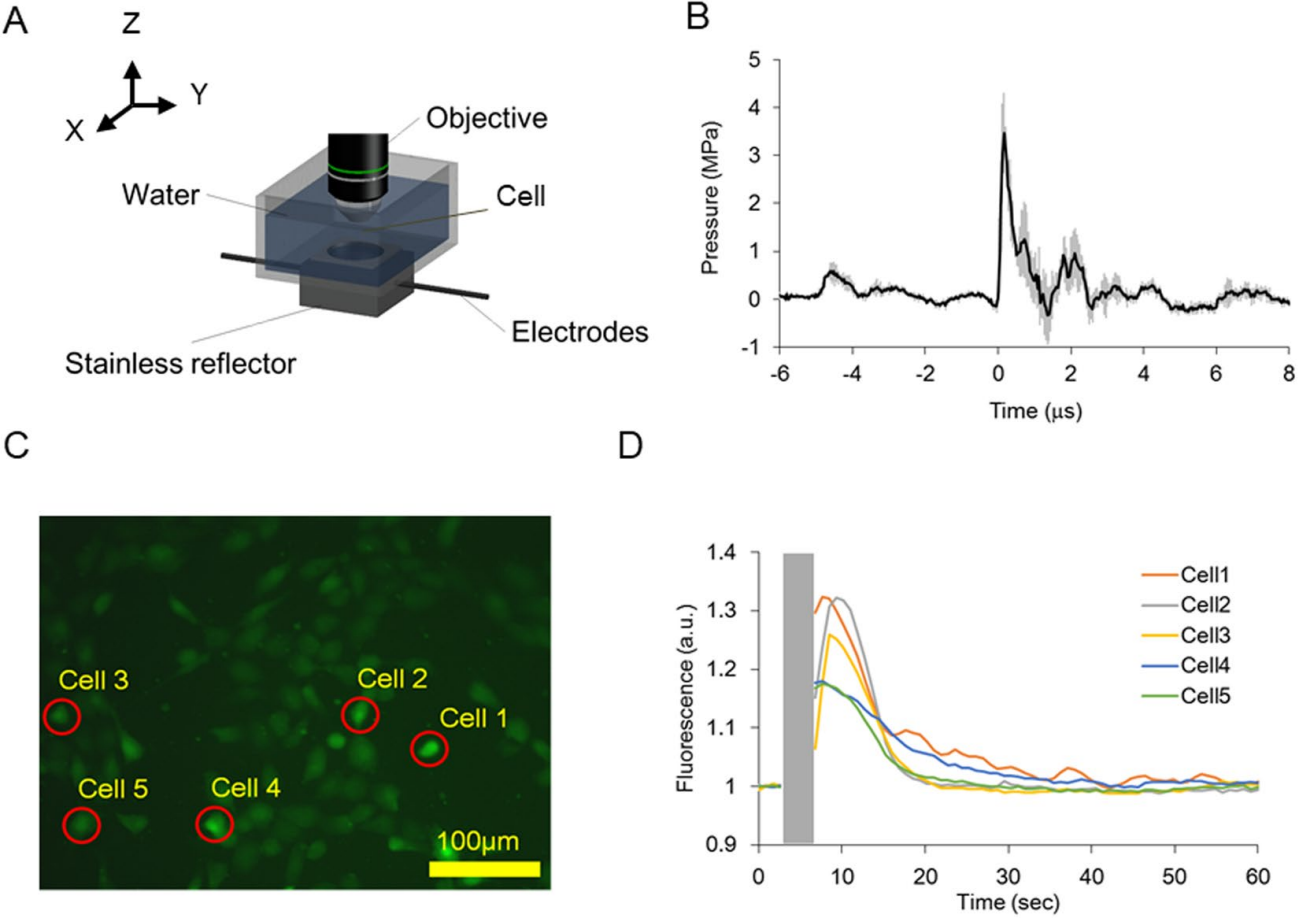
LESW irradiation and intracellular Ca^2+^ increase. (**A**) LESWs were generated among electrodes and refocused with a stainless reflector. An objective was set when an intracellular Ca^2+^ increase was measured. See Supplementary Fig. S1A for detail. (**B**) A pressure profile of LESWs with a discharge voltage of 3 kV (means ± SEMs, N = 4). (**C**) A fluorescence image obtained with the objective. Red circles around cells 1–5 indicate cells 1–5 in **D**. (**D**) Intracellular Ca^2+^ increase evoked by a single-shot LESW with an acoustic energy of 1.7 μJ/mm^2^.

Shock waves generated by high-voltage discharge in water spread spherically. The spread shock waves were refocused with a stainless reflector to irradiate the shock waves with sufficient acoustic energies (Fig. 1A, Supplementary S1A). Around the refocus point, acoustic energy was supposed to be distributed non-uniformly in a spatial manner. To obtain a spatial distribution of acoustic energy, peak pressures of shock waves were measured along the X- and Y-axes, which were set perpendicular to the shock-wave transmission (Fig. 1A, Supplementary S1A). When the discharged voltage was 3 kV, the peak pressure of shock waves at the refocus point was 3.8 ± 0.5 MPa (N = 4, Fig. 1B). Depending on the distances from the refocus point, peak pressures decreased gradually along the two axes (Supplementary Fig. S1B,C). Close to the refocus point, peak pressures were distributed rather uniformly. To measure the width of the region in which peak pressures are distributed uniformly, the regions in which peak pressures were within −3 dB were estimated along the two axes. Along the X- and Y-axes, the regions with uniform peak pressures distributed were 2.7 mm and 3.5 mm wide, respectively. This result shows that shock waves were irradiated with uniform acoustic energies around the refocus point in a region within 2.7 mm.

Intracellular Ca^2+^ increase was observed in bovine aortic endothelial cells (BAECs) positioned at the refocus point. Endothelial cells are one of mechanosensitive cell types^18,20,22^. The imaging region for fluorescence imaging was 0.46 × 0.35 mm (Fig. 1C). This was smaller than the region with uniform acoustic energies, within 2.7 mm around the refocus point. Thus, in the imaging region, all the cells were irradiated with uniform acoustic energies. The acoustic energy estimated by integrating the square of pressure values was 1.7 ± 0.6 μJ/mm^2^ (N = 4). Irradiation of single-shot shock waves on BAECs with the uniform acoustic energy evoked intracellular Ca^2+^ increases (Fig. 1D). Among the cells in the imaging region, the fraction of cells that evoked intracellular Ca^2+^ increases was 39 ± 9% (N = 7).

### Minimum energy intensity of LESWs sufficient for evoking intracellular Ca^2^ increase was 1.7 μJ/mm^2^

**Figure 2:**
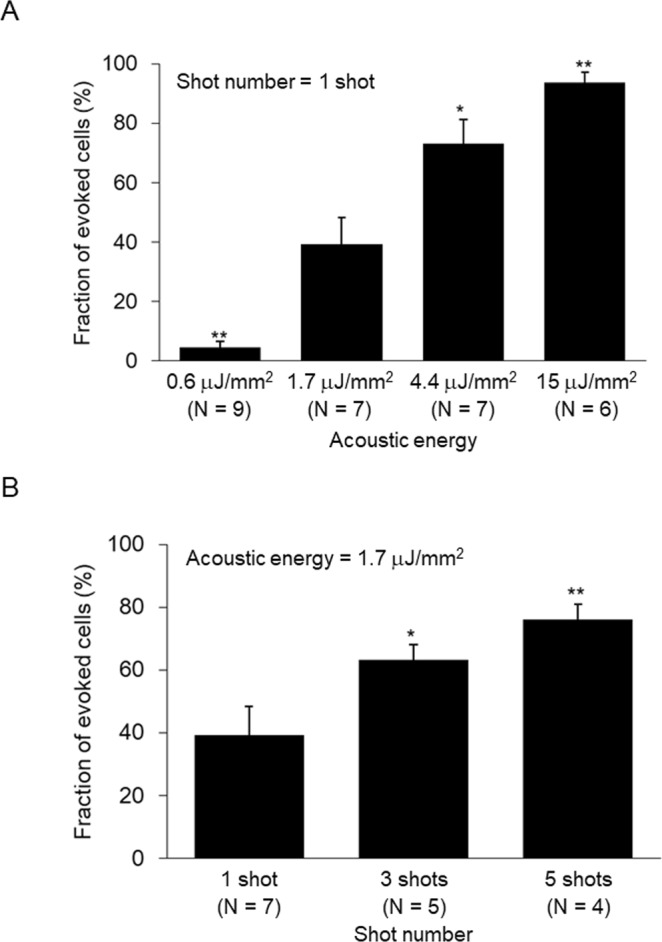
Dependence of intracellular Ca^2+^ increase on acoustic energy (**A**) and shot number (**B**).

Suppressed acoustic energy is more likely to evoke an intracellular Ca^2+^ increase without inducing sonoporation. The acoustic energy of shock waves was suppressed by inserting an acrylic plate between the stainless reflector and the refocus point (Supplementary Fig. S2A). At the refocus point, acoustic energy was suppressed to 0.6 ± 0.2 μJ/mm^2^ (N = 4, Supplementary Fig. S2B,E,F). With the suppressed acoustic energy, the fraction of cells that evoked an intracellular Ca^2+^ increase was limited to 4.5 ± 2.1% (N = 9, Fig. 2A). Although acoustic energy of 0.6 μJ/mm^2^ can evoke an intracellular Ca^2+^ increase, acoustic energy of 1.7 μJ/mm^2^ was revealed to be sufficient. Next, acoustic energy was increased by increasing the discharge voltage. When the discharge voltage was increased to 4 and 5 kV, acoustic energy increased to 4.4 ± 0.1 and 15.8 ± 6 μJ/mm^2^, respectively (N = 4, Supplementary Fig. S2C–F). With these increased acoustic energies, the fractions of evoked cells increased to 73 ± 8 and 93 ± 4%, respectively (N = 7 and 6, Fig. 2A). This result shows that the evocation of an intracellular Ca^2+^ increase depends on acoustic energy. The minimum acoustic energy required for sufficient evocation was 1.7 μJ/mm^2^.

Shot number was another parameter for shock wave irradiations. Shock waves with an acoustic energy of 1.7 μJ/mm^2^ were irradiated on cells with increased shot numbers; 3 and 5 shots (1 Hz). When the shot number was increased to 3 and 5, the fraction of evoked cells increased to 63 ± 5 and 76 ± 5%, respectively (N = 5 and 4, Fig. 2B). This result shows that the evocation of an intracellular Ca^2+^ increase depends on the number of shots. Suppressed mechanical loading is supposed to provide an advantage in suppressing sonoporation. One shot was ideal for evoking an intracellular Ca^2+^ increase.

### Sonoporation was not observed with single-shot 1.7 μJ/mm^2^ LESWs

**Figure 3:**
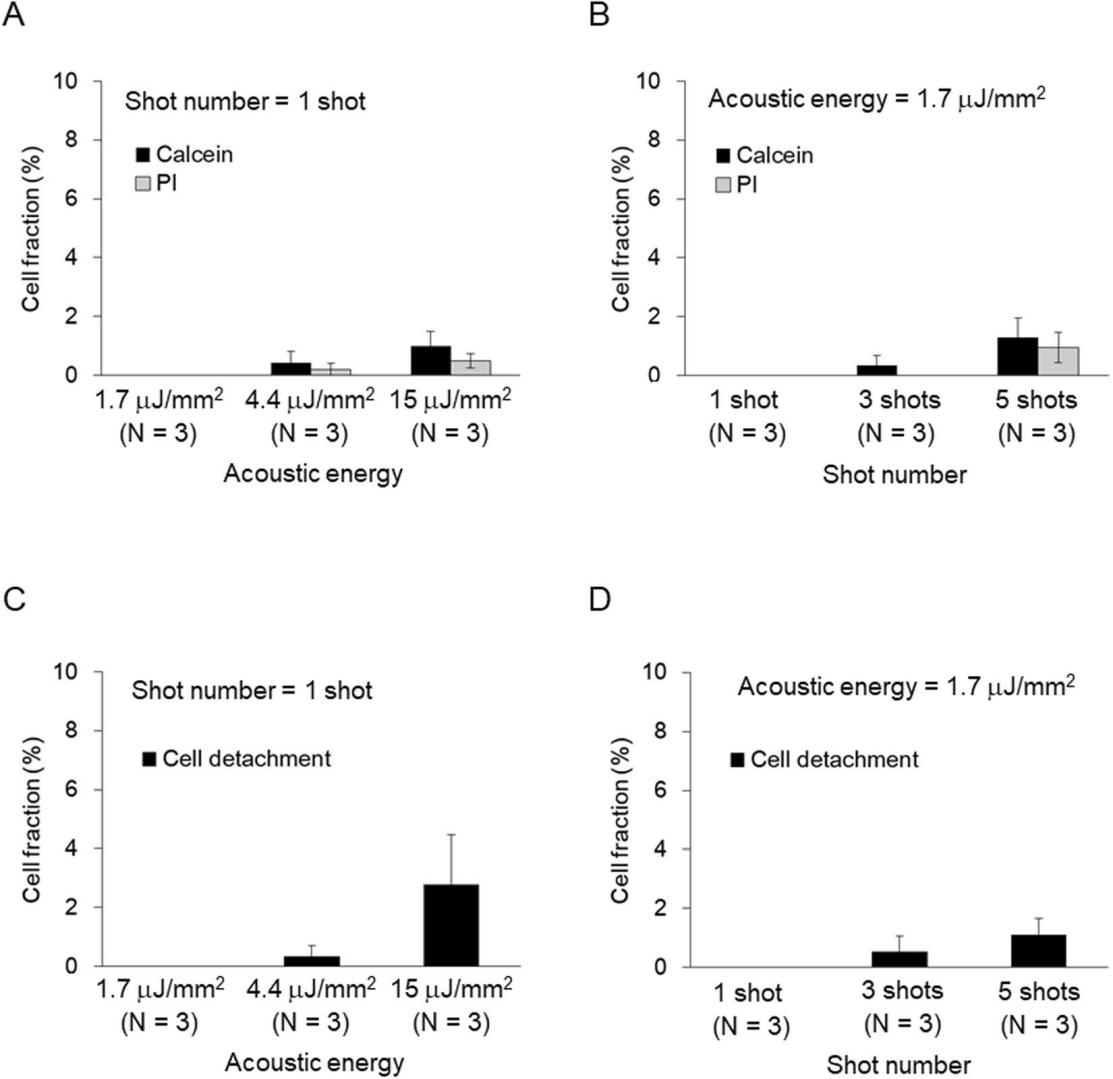
Pore formation on plasma membrane and cell detachment. Fraction of cells permeated by calcein and PI was dependent on acoustic energy (**A**) and shot number (**B**). Fraction of detached cells was dependent on acoustic energy (**C**) and shot number (**D**). In each sample, 107 ± 7 cells (mean ± SEM, 15 samples) were analyzed.

The minimal acoustic energy of shock waves to evoke a sufficient intracellular Ca^2+^ increase was 1.7 μJ/mm^2^. To investigate the suppression of sonoporation with the minimal acoustic energy, pore formations were investigated with calcein leakage and propidium iodide (PI) influx. The Stokes radii of calcein and PI are 0.6–0.7 nm^23,24^. When cells were irradiated with the minimal acoustic energy, neither calcein leakage nor propidium iodide influx was observed. When the acoustic energy was increased to 4.4 or 15.8 μJ/mm^2^, both calcein leakage and PI influx were observed. However, the fraction was limited to less than 2% of total cells (Fig. 3A). When the number of shots was increased to 3 or 5, both calcein leakage and PI influx were also observed. The fraction of cells was still limited to under 2% (Fig. 3B). With the minimal acoustic energy, single-shot shock waves failed to induce sonoporation. These results show that LESWs can evoke intracellular Ca^2+^ increases independently of sonoporation.

### Cell detachment was not observed with single-shot 1.7 μJ/mm^2^ LESWs

Shock waves can detach cells^25,26^. Cell detachments evoke an intracellular Ca^2+^ increase in surrounding cells^27^. It is also possible that intracellular Ca^2+^ increases were evoked by cell detachment. To understand whether cell detachment was involved in the intracellular Ca^2+^ increase, fractions of cell detachment were quantified. Single-shot shock waves with acoustic energy of 1.7 μJ/mm^2^ failed to detach cells. When acoustic energy was increased to 4.4 or 15.8 μJ/mm^2^, cell detachments were observed in a limited fraction of cells, less than 3% (Fig. 3C). When the number of shots was increased to 3 or 5, cell detachments were also observed, but the fraction was limited to less than 1% (Fig. 3D). With acoustic energy of 1.7 μJ/mm^2^, cell detachment can be excluded. These results show that LESWs can evoke intracellular Ca^2+^ increases independently of cell detachment.

### Actin cytoskeletons are involved in the intracellular Ca^2+^ increase

**Figure 4:**
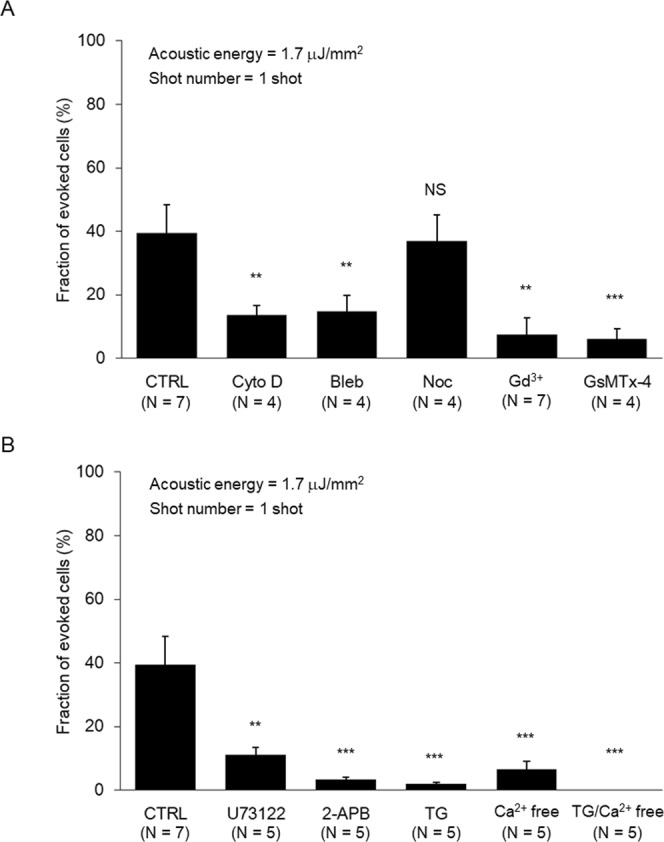
Subcellular structure and Ca^2+^ cascades involved in the intracellular Ca^2+^ increase. (**A**) Inhibitions by CytoD and Bleb show the involvement of actin cytoskeletons. Inhibitions by Gd^3+^ and GsMTx-4 show the involvement of SA channels. (**B**) Inhibition by U73122, 2-APB, and TG show the involvement of Ca^2+^ release via the PLC-IP^3^-IP_3_R cascade. Inhibition by depleting extracellular Ca^2+^ with Ca^2+^-free solution show the involvement of Ca^2+^ influx.

Previous studies showed that caveolin and VE-cadherin are involved in cellular signals evoked by LESWs^11,12^. Both caveolae, which caveolins compose, and adherens junctions, which VE-cadherin compose, connect with actin cytoskeletons^28,29^. Thus, the involvement of actin cytoskeleton in LESW mechanosensing is reasonable. To understand whether an intracellular Ca^2+^ increase evoked by LESWs involves cytoskeletons, actin cytoskeletons were first inhibited. Polymerization and the contraction force of actin cytoskeletons were inhibited by cytochalasin D (CytoD) and blebbistatin, respectively. With the inhibitions, the intracellular Ca^2+^ increase was inhibited (Fig. 4A). Thus, the intracellular Ca^2+^ increase involved actin cytoskeletons.

Some mechanical stimulation involves both actin cytoskeletons and microtubules in mechanosensing^22,30,31^. Then, the polymerization of microtubules was inhibited by nocodazole, but the intracellular Ca^2+^ increase was not inhibited. Thus, the intracellular Ca^2+^ increase did not involve microtubules (Fig. 4A). These results indicate that the intracellular Ca^2+^ increase evoked by LESWs involves a part of cytoskeletons. Although actin cytoskeletons are involved, microtubules are not. However, some others do not involve microtubules^32–35^. Thus, it is probable that LESWs evoke the intracellular Ca^2+^ increase without involving microtubules.

### SAChs are involved in the intracellular Ca^2+^ increase

Shock wave irradiations induce structural changes in the lipid bilayer^36^. Alteration of tension in plasma membrane can activate SACh. Thus, SACh is another cellular component that could be involved in cellular activations induced by LESWs. To understand whether the intracellular Ca^2+^ increase by LESWs involves SAChs, SAChs were inhibited with Gd^3+^ and GsMTx-4^18^. This inhibited the intracellular Ca^2+^ increase (Fig. 4A). Thus, along with actin cytoskeletons, SAChs are also involved in the intracellular Ca^2+^ increase evoked by LESWs.

### Both Ca^2+^ release and Ca^2+^ influx are involved in the intracellular Ca^2+^ increase

Mechanical stimulations evoke the intracellular Ca^2+^ increase with two typical Ca^2+^ cascades. One is Ca^2+^ release from the stored intracellular Ca^2+^ following the PLC-IP_3_-IP_3_R cascade^16,17^. Another is Ca^2+^ influx from extracellular fluid through Ca^2+^ channels. First, to understand whether the intracellular Ca^2+^ increase evoked by LESWs involves Ca^2+^ release, PLC and IP_3_R were inhibited with U73122 and 2-APB, respectively. Furthermore, the store of intracellular Ca^2+^ was depleted by thapsigargin. These inhibitions inhibited the intracellular Ca^2+^ increase (Fig. 4B). Thus, the intracellular Ca^2+^ increase involves Ca^2+^ release from the store of intracellular Ca^2+^. Next, to understand whether the intracellular Ca^2+^ increase involves Ca^2+^ influx, extracellular Ca^2+^ was depleted. This also inhibited the intracellular Ca^2+^ increase (Fig. 4B). Thus, the intracellular Ca^2+^ increase also involves extracellular Ca^2+^ influx. These results indicate that the intracellular Ca^2+^ increase evoked by LESWs involves both Ca^2+^ release and Ca^2+^ influx as well as other mechanical stimulations.

## Discussion

LESWs evoked an intracellular Ca^2+^ increase in endothelial cells. The minimum acoustic energy required for the evocation was 1.7 μJ/mm^2^. With the minimum acoustic energy, LESWs failed to induce sonoporation. Actin cytoskeleton and SA channel were involved in the intracellular Ca^2+^ increase. Both Ca^2+^ influx through the Ca^2+^ channels and Ca^2+^ release through the PLC-IP_3_-IP_3_R cascade were involved in this increase. These results show that LESWs evoke the intracellular Ca^2+^ increase through mechanosensing rather than sonoporation.

The acoustic energies of shock waves used for tissue healing and cellular activation have been in the range of 30–400 μJ/mm^2–12^. In previous studies, cellular responses were detected as a result of enhancements of gene expression and protein synthesis. In those detections, cellular responses in large numbers of cells were required. In this study, cellular responses were detected as a result of an intracellular Ca^2+^ increase. This detection requires cellular responses in single cells. By detecting an intracellular Ca^2+^ increase, cellular responses were detected accompanying acoustic energies in the range of 0.6–15.8 μJ/mm^2^ (Fig. 2A). With the suppressed acoustic energy, the cellular response evoked was independent of sonoporation. Furthermore, with a cellular response that is independent of sonoporation, underlying cascades could be investigated.

The threshold for sonoporation by shock waves has been discussed^15^. Among physical parameters for shock waves, impulse was suggested to be important. A shock wave impulse can be written as I = P_0_·Δt, where P_0_ and Δt are peak pressure and impulse half width, respectively. It was suggested that the threshold for sonoporation is around 4 Pas^15^. In the present study, sonoporation was observed with shock waves having an acoustic energy of 4.4 μJ/mm^2^ (Fig. 3A). The impulse of the shock waves was calculated to be 4.1 ± 0.8 Pas (N = 4). Thus, the threshold of impulse required for sonoporation was analogous to that in the previous study. Intracellular Ca^2+^ increase was evoked even with a lower acoustic energy of 0.6 μJ/mm^2^, *i.e*., with an impulse of 1.5 ± 0.4 Pas (N = 4). Thus, LESWs are supposed to evoke an intracellular Ca^2+^ increase under the threshold for sonoporation.

The mechanisms underlying the involvement of actin cytoskeletons and SAChs in the intracellular Ca^2+^ increase remain elusive. Shock waves largely deform cells during their tensile phase, not during the compression phase^37^. In the present study, although the compression phase was dominant, the tensile phase was also slightly observed (Fig. 1B). Thus, the physical mechanisms can be deformation under the tensile phase. The pressure gradient during pressure increment and following decrement can generate forces in cells^38^. These mechanical forces can enforce actin cytoskeleton and cellular components connected to actin cytoskeleton. When cytoskeleton is enforced, physical forces transmit through cellular junction and cellular adhesion. In those cellular components, mechanosensors such as cadherin and integrin are finally enforced. Forces by magnetic beads applied to actin cytoskeleton transmit through focal adhesion and activate SACh close to the focal adhesion^22,30^. Thus, forces applied to cytoskeletons can activate SAChs. Enforced membranes can also activate SAChs.

Another possible mechanism is the attenuation of acoustic energies by actin cytoskeleton^39^. Following such attenuation, those energies are used to dissociate crosslinkers on actin cytoskeletons^40^. This dissociation might alter the physical balance in actin cytoskeleton. Either the imbalance or dissociation itself can induce biological signaling^41,42^. The main frequency estimated from pressure profiles of LESW was around 0.3 MHz in the present study. Attenuation of ultrasound by cells is 0.021 Neper/mm/MHz^43^. Thus, assuming that shock wave attenuation is analogous to ultrasound attenuation^44^ and that the cellular thickness is 5 μm, shock wave attenuation efficiency by cells is estimated to be 5.3 × 10^−5^ Neper. This means that 0.0053% of acoustic energy would be attenuated. With a peak pressure of 3.8 MPa, energy flux is estimated to be 1.7 μJ/mm^2^. The BAECs had a cellular region area of 3.9 × 10^−4^ mm^2^ (N = 443). For each BAEC, 0.66 nJ of energy flux transmitted. Thus, the attenuated acoustic energy per single-shot LESW is estimated to be 0.035 pJ. This level of energy is analogous to that required to dissociate 2% of crosslinkers^40^.

Blast shock waves are generated by explosives. As a model of blast injury, the intracellular Ca^2+^ increase evoked in astrocytes by blast shock waves was investigated^45,46^. The peak pressure of the blast shock waves was relatively comparable: around 1 MPa for blast shock waves and typically 3.8 MPa for shock waves in this study (Fig. 1B). However, the pulse width of blast shock waves was several hundred microseconds for blast shock waves and typically 1 μs for shock waves in this study. Shock waves with physical parameters having large difference can induce different physical effects on cells. Although intracellular Ca^2+^ increases are commonly evoked by blast shock waves and LESWs, the underlying physical and biological mechanisms might be better investigated independently.

In summary, LESWs evoked intracellular Ca^2+^ increases. The minimum energy flux required for evocation was 1.7 μJ/mm^2^ when the shot number was limited to one. With the minimized energy fluxes, LESWs failed to form transient pores on plasma membrane through which calcein and PI transfer. Actin cytoskeletons and Ca^2+^ channels were involved in the intracellular Ca^2+^ increase. Cascades for intracellular Ca^2+^ increases were Ca^2+^ released through the PLC-IP_3_-IP_3_R pathway and Ca^2+^ influx through plasma membrane. These results show that LESWs can evoke mechanosensing independently of sonoporation.

## Materials and Methods

### Shock wave generation

Between two electrodes (tungsten carbide, φ  = 0.5, a gap of 200 µm), high voltages of 3–5 kV were applied by a voltage power supply (Sparkling Photon)^47^. The applied high voltages were suddenly discharged in water, thus generating shock waves. Water surrounding the electrodes was circulated because the discharges generated bubbles. Using a stainless reflector, the generated shock waves were refocused^47^. The reflector had a spheroid surface. The distance between the foci on the spheroid surface was 22.4 mm. Due to the large difference between the acoustic impedances of stainless and water, *i.e*., 4.7 × 10^7^ kg/m^2^s and 1.5 × 10^6^ kg/m^2^s, the reflection coefficient was expected to reach as high as 94%. When the supplied voltages were below 3 kV, the discharge failed to occur. To generate shock waves with lower peak pressures, an acrylic plate (t = 1 mm) was inserted between the foci.

### Pressure measurement

Pressure profiles were measured with a polyvinylidene difluoride (PVDF) needle hydrophone with a sensitive diameter of 0.5 mm (Müller-Platte Needle Probe, Dr. Müller Instruments). The point of origin was set at the second focus of the spheroid reflector. The Z-axis was set along the direction of shock wave propagation. Parallel to the electrodes, the Y-axis was set. The X-axis was set so that it crossed the Z- and Y- axes orthogonally (Fig. 1A, S1A). To obtain the spatial distribution of peak pressures, peak pressures were measured 10 times at each measurement point. Along the Z-axis, peak pressures were measured every 2 mm from 0 mm to + 10 mm. Along the X- and Y-axes, peak pressures were measured every 0.5 mm from −3 mm to + 3 mm. When peak pressures were measured, a polystyrene plate (t = 0.2 mm) was inserted in a plane at Z = −0.5 mm to mimic the condition when a cell chamber was set. At the point of origin, the pressure profiles of shock waves were also obtained. The energy fluxes of the shock waves were estimated by integrating the square of the pressure values. Impulses were estimated by integrating the pressure values.

### Cell chamber

Polystyrene sheets, 200 µm thick and 10 × 10 mm in area, were sonicated with 99.5% ethanol for 5 min and dried under a nitrogen flow. A silicone rubber wall, 6 mm in inner diameter and 1 mm thick, was attached to the polystyrene plate and treated with oxygen plasma (PDC-32G, Harrick Plasma) for 30 sec. After sterilization with 70% ethanol under UV irradiation for 20 min, the cell chambers were incubated with collagen solution (50 μg/ml in PBS, Koken) for 1 h and washed with PBS three times. The acoustic impedance of polystyrene is 2.4 × 10^6^ kg/m^2^s and comparable to that of water, 1.5 × 10^6^ kg/m^2^s. This similarity was expected to transmit LESWs into cell chambers with high efficiency. At the second focus, a cell chamber was set for shock wave irradiations.

### Fluorescence imaging

BAECs (Lonza) were cultured in Dulbecco’s Modified Eagle’s Medium (DMEM, Sigma) supplemented with 10% fetal bovine serum, 1% penicillin-streptomycin (Gibco), and 2 mM L-glutamine (Sigma). After the cells reached confluence, they were detached and seeded in the cell chambers. For 1–2 days before the experiments, the cells were cultured in an incubator at 37 °C with 100% humidity of 5% CO_2_. Cells were washed with HEPES buffered saline solution (HBSS (in mM): NaCl 130, KCl 5.4, CaCl_2_ 1.8, MgCl_2_ 0.8, glucose 5.5, HEPES 20; pH adjusted to 7.4 with NaOH) and loaded with Fluo-4AM (2 µM in HBSS, 0.04% pluronic, Dojindo) for 30 min at room temperature. The cells were washed three times and stabilized at room temperature for 30 min. After the cell chamber was covered with a polyvinylidene chloride film 11 μm thick, the chamber was set on a plane at Z = −0.5 mm close to the second focus of the stainless reflector (Supplementary Fig. S3A,B).

With an upright microscope (BX51WI, Olympus), excitation light having a wavelength of 490 nm was irradiated, and emission light having a wavelength of 520 nm was corrected through an objective lens (20×, N.A 0.50, Olympus). Fluorescence images were obtained with a digital 3CCD camera (ORCA-3CCD, Hamamatsu) with a sampling rate of 0.3–0.5 seconds. When shock waves were irradiated on cells, the objective lens was displaced out of the water for 3–5 seconds to prevent shock wave reflection at the lens surface. During shock wave irradiations, cell chambers were maintained at 37 °C.

The fluorescence intensity in each cell was quantified with image analysis software (Aquacosmos, Hamamatsu Photonics). The time courses of fluorescence intensities were divided by the fluorescence intensities before the shock wave irradiations. When the ratio exceeded 1.1, it was considered to have reacted. Cellular regions were calculated by using the particle analyze command in ImageJ (NIH).

### Inhibition experiments

After loading Fluo-4AM onto cells, the cells were loaded with inhibitors. To inhibit Ca^2+^ release, either thapsigargin (1 µM in HBSS, Sigma), U73122 (1 μM in HBSS, Calbiochem), or 2-APB (1 µM in HBSS, Sigma) was loaded for 30 min. To inhibit Ca^2+^ influx, extracellular Ca^2+^ was depleted with Ca^2+^-free HBSS (NaCl 130, KCl 5.4, MgCl_2_ 2.6, glucose 5.5, HEPES 20, EGTA 0.1; in mM)^21^ for 30 min. To inhibit SAChs, either Gd^3+^ (1 µM in HBSS, Sigma) or GsMTx-4 (1 µM in HBSS, Sigma) was loaded. To inhibit cytoskeletons, either cytochalasin D (10 μM in HBSS, Sigma), blebbistatin (10 μM in HBSS, Sigma), or nocodazole (1 μM in HBSS, Sigma) was loaded for 30 min. Without washing away the inhibitors, the cells were irradiated with shock waves.

### Plasma membrane permeabilization and cell detachment

Cell membrane permeabilization was analyzed by measuring calcein leakage from cytosol. Cells were loaded with calcein AM (0.5 µM in HBSS, Dojindo) for 30 min and stabilized at room temperature for 30 min. Fluorescence images were obtained with a 3CCD camera 0.3–0.5 sec intervals. Plasma membrane permeabilization was detected by fluorescent decay. Fluorescence intensities in single cells at 6 min after shock wave irradiation were normalized with those from before shock wave irradiation. From the normalized fluorescence intensities, average intensity $\bar{x}$ and standard deviation *σ* were calculated in each image. Plasma membranes were determined to be permeabilized if the normalized fluorescents were less than $\bar{x}-3\sigma$. Cell detachment was determined by a sudden disappearance of fluorescence.

### Statistical analysis

Data are expressed as the means ± SEMs (standard error of the mean). Differences between groups were calculated by paired Student’s t-test. Statistical significance is indicated by the symbol “*” when p is below 0.05 and “**” when p is below 0.01.

## Supplementary information

Supplemental Information

## Data Availability

The datasets generated during and/or analysed during the current study are available from the corresponding author on reasonable request.
