# Supplementary material for: Low-energy shock waves evoke intracellular Ca2+ increases independently of sonoporation
Source: Sci Rep. 2019 Mar 1;9:3218. doi: 10.1038/s41598-019-39806-x (PMC6397190; doi:10.1038/s41598-019-39806-x)
Supplement: Supplementary file 1 — Supplemental Information [file 41598_2019_39806_MOESM1_ESM.pdf]

# Low-energy shock waves evoke intracellular $Ca^{2+}$ increases independently of sonoporation

Toru Takahashi[1], Keiichi Nakagawa[2], Shigeru Tada[1], Akira Tsukamoto[1,*]

[1]Department of Applied Physics, Graduate School of Science and Engineering, National Defense Academy, Hashirimizu 1-10-20, Yokosuka, Kanagawa 239-8686 Japan

[2]Department of Precise Engineering, Graduate School of Engineering, The University of Tokyo, Hongo 7-3-1, Bunkyo-ku, Tokyo 113-8656 Japan

*Email: tsuka@nda.ac.jp

**SUPPLEMENTARY FIGURES**

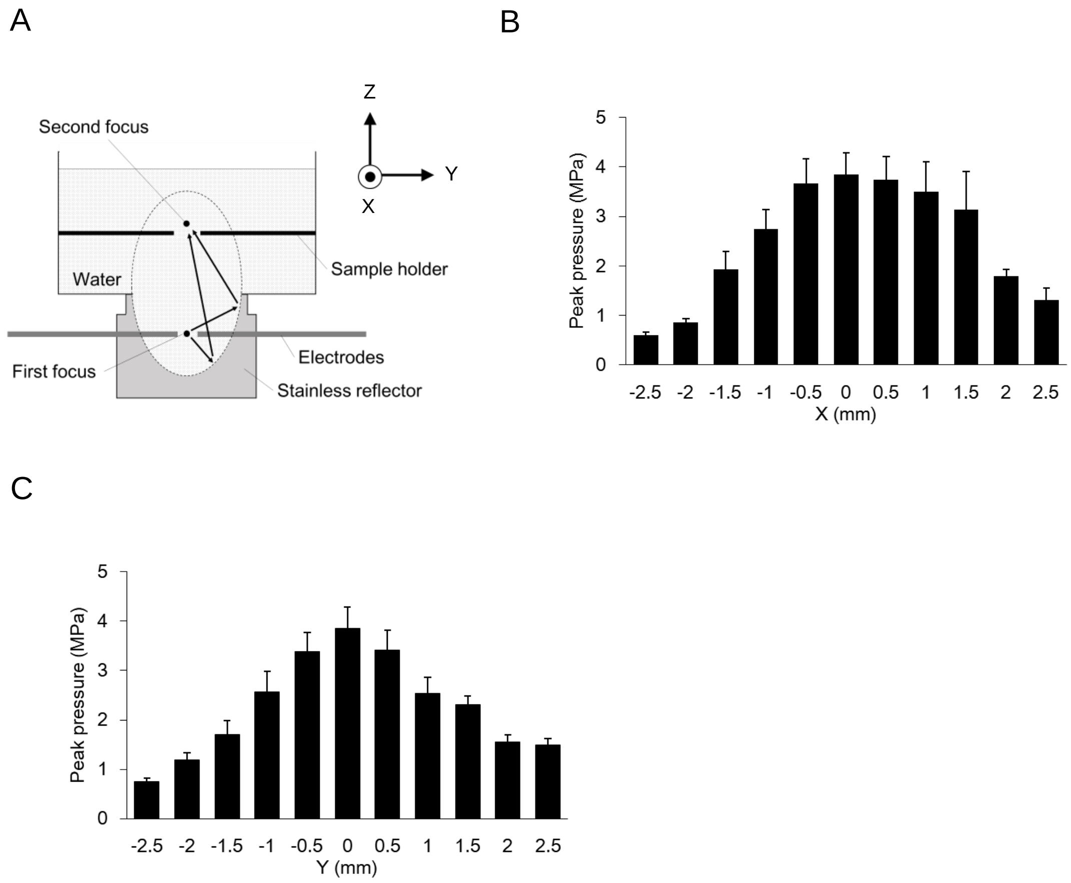


**Supplementary Figure S1.** Peak pressure distribution along X- and Y-axes. (A) Shock waves were generated at the first focus on the spheroidal surface of a stainless reflector,. Following the reflection, shock waves were refocused to the second focus. The X- and Y-axes were set perpendicular shock wave transmission. Peak pressure distributions along the X-axis (B) and Y-axis (C) show uniform distribution close to the second focus.

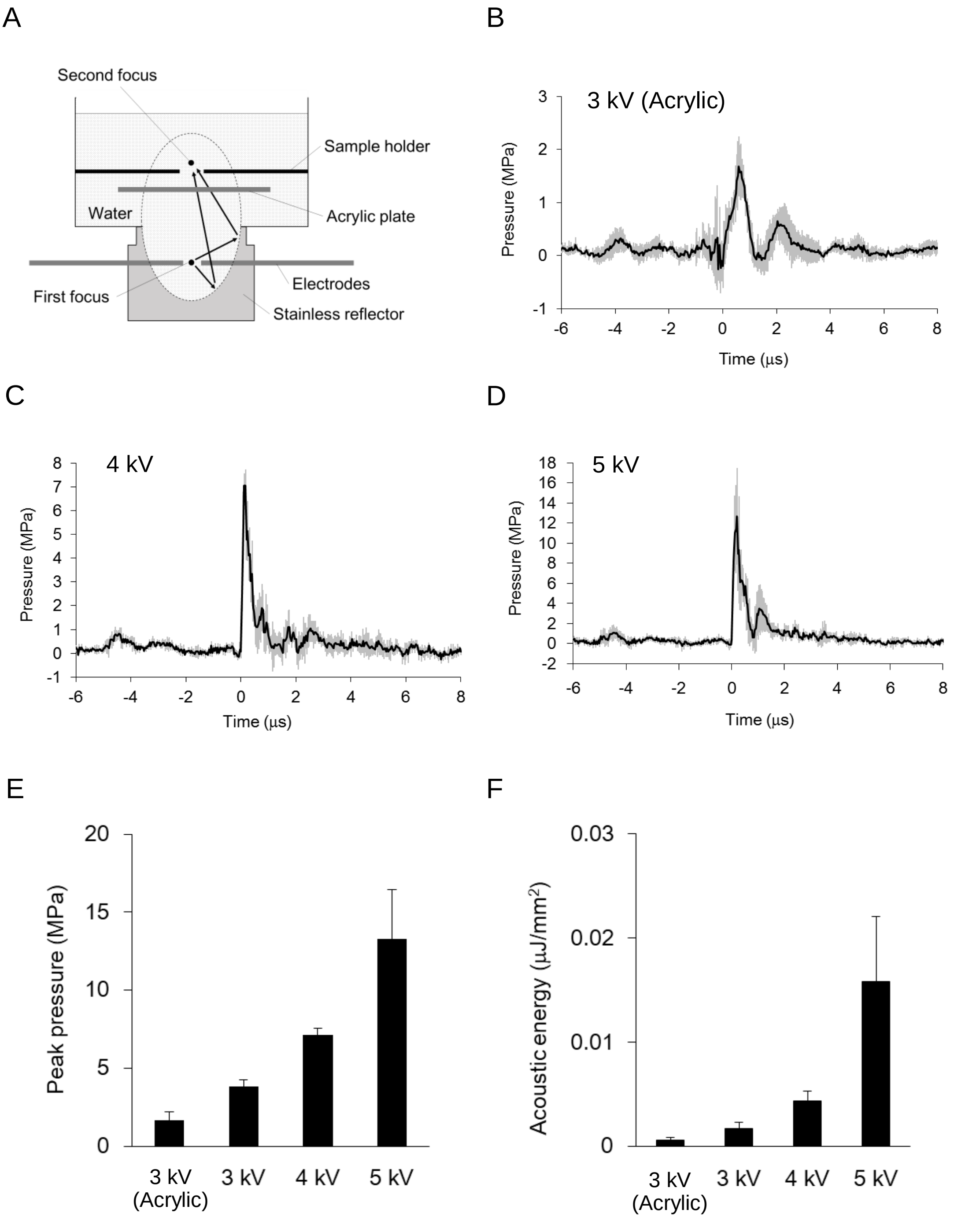
A
B
C
D
E
F
Second focus
Sample holder
Acrylic plate
Water
Electrodes
First focus
Stainless reflector
3 kV (Acrylic)
4 kV
5 kV
Pressure (MPa)
Time (μs)
Peak pressure (MPa)
Acoustic energy (μJ/mm2)
3 kV (Acrylic)
3 kV
4 kV
5 kV

**Supplementary Figure S2.** Dependence of peak pressure and acoustic energy on shock wave irradiation conditions. (A) Peak pressure and acoustic energy were suppressed by inserting an acrylic plate. (B) A pressure profile of LESWs having a discharge voltage of 3 kV with the inserted acrylic plate (means ± SEMs, N = 4). (C) Pressure profile of LESWs with a discharge voltage of 4 kV (means ± SEMs, N = 4). (D) A pressure profile of LESWs with a discharge voltage of 5 kV (means ± SEMs, N = 4). Peak pressure (E) and acoustic energy (F) of shock waves generated and transmitted under different conditions.

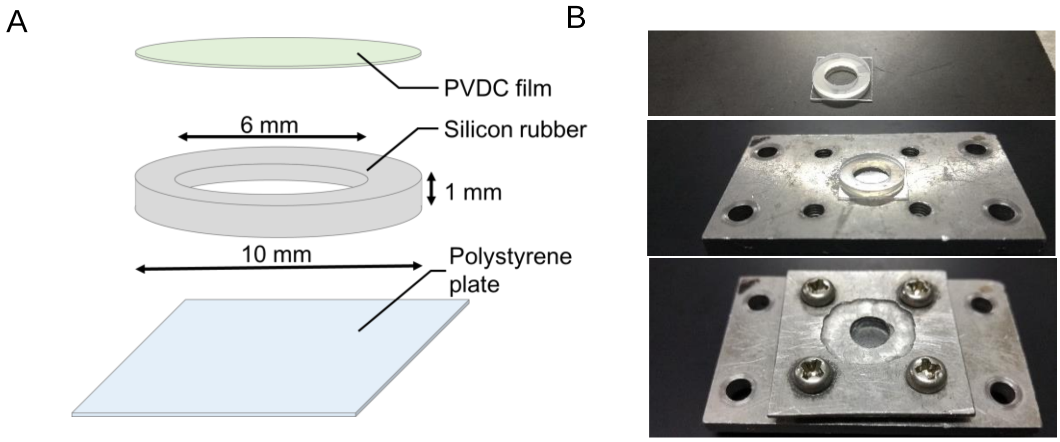


**Supplementary Figure S3.** Cell chamber and its holders. (A) Assembly of cell chamber. (B) Combining cell chamber with its holder before setting in the sonication chamber.
